# Constructing a Multidisciplinary Network That Relies on Disruptive Technologies to Design, Test, and Implement Simulation Training

**DOI:** 10.7759/cureus.7548

**Published:** 2020-04-05

**Authors:** Krystina M Clarke, Bill Kapralos, Alvaro Quevedo, Adam Dubrowski

**Affiliations:** 1 Health Sciences, Ontario Tech University, Oshawa, CAN; 2 Faculty of Information Technology/Health Education Technology Research Unit, Ontario Institute of Technology, Oshawa, CAN; 3 Faculty of Business and Information Technology, Ontario Tech University, Oshawa, CAN

**Keywords:** simulation, disruptive technologies, 3d printing, extended reality, game development, multidisciplinary, interprofessional collaboration, health professions education

## Abstract

MaxSIMhealth is a multidisciplinary network of manufacturing, design, and simulation labs at Ontario Tech University combining expertise in health sciences, business and information technology (IT), and engineering while building community partnerships to advance simulation training. It discovers existing simulation gaps, provides innovative solutions that change systems, and leads to improved healthcare outcomes. Specifically, it utilizes disruptive technologies, including 3D printing, gaming, and extended reality, as innovative solutions that deliver cost-effective, portable, and realistic simulation, which is currently lacking. MaxSIMhealth is a novel collaborative innovation with aims to develop future cohorts of scholars with strong competencies ranging from technology application, to collaborating in new environments, communicating professionally, and problem-solving. Its work will transform current health professional education landscapes by providing novel, flexible, and inexpensive simulation environments. This editorial aims to showcase maxSIMhealth's innovative strategy focusing on collaborations of expertise in order to develop new simulation solutions that advance the health industry.

## Editorial

Background

Simulation combines different technologies and techniques to create an imitation or amplification of a real event in order to learn, practice, evaluate, test, or understand systems or human actions [1]. Simulation can be virtual, physical, or a mixture of both and must be contextually accurate in order to be efficacious and effective. Therefore, when designing simulation experiences, a single profession (e.g. computer science) does not hold the answer as to how to build the experiential and educational event, but instead several professions need to work together for optimal results. This editorial aims to showcase maxSIMhealth's innovative strategy focusing on expert collaboration in order to develop new simulation solutions that could improve health outcomes.

Innovation

MaxSIMhealth (maxSIMhealth.com) is a synergistic multidisciplinary network whose aim is to design simulation experiences. This is made possible because it is an academic public-for-profit network based out of Ontario Tech University with access to several different manufacturing, design, and simulation labs. It is supported by a blended funding model with institutional support, with funding through National funding agencies, including Canadian Foundation for Innovation, Canada Research Chair in HealthCare Simulation (through Canadian Institute for Health Research), Natural Sciences and Engineering Research Council, and Social Sciences and Humanities Research Council. 

**Figure 1:**
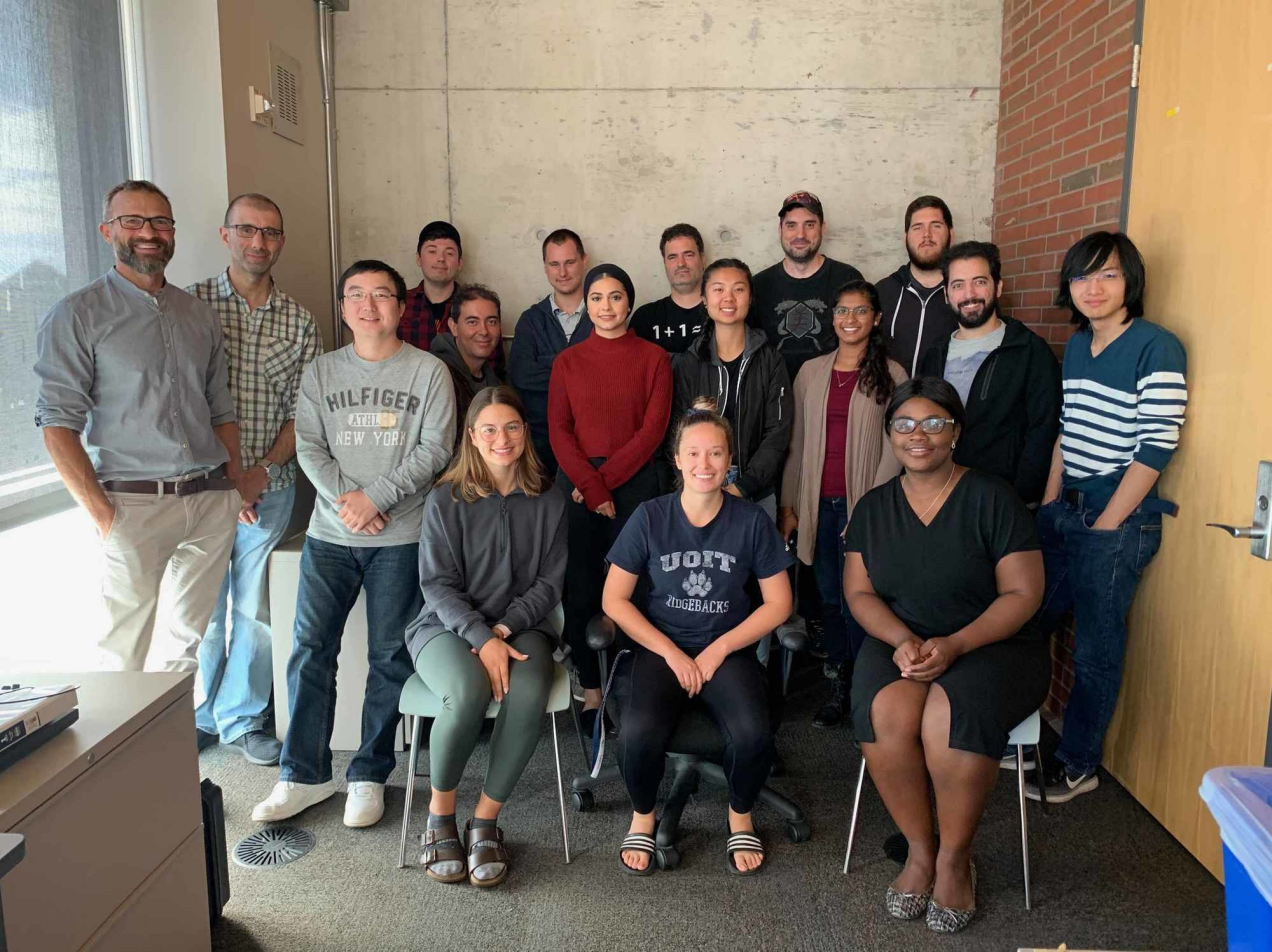
The maxSIMhealth team. Pictured above are the many individuals who form the collaborative system. Within this group are undergraduate, graduate, PhD and postdoctoral students, professors, and researchers. Each individual comes from a unique academic and work background. Team members of maxSIMhealth are part of the Faculties of Health Sciences, Business and IT, Engineering and Applied Sciences, and more which plays a key role in the unique functionality and perspectives the group holds.

MaxSIMhealth combines expertise in faculties across the university, including health sciences, business and IT (computer science, game development, etc.), engineering and applied sciences, education, and social sciences (see Figure 1). Furthermore, the collaborative builds on existing and new community partnerships: Lakeridge Health Hospital, Durham Region Department of Health, Canadian Society for Medical Laboratory Sciences, and Simulation Canada. In addition, maxSIMhealth actively seeks and establishes research partnerships with not-for-profit and for-profit organizations as commercial channel partners and stakeholders in order to advance simulation training globally. Finally, it acts as an idea-seeding mechanism for a local incubator system, Brilliant Catalyst. 

This multisectoral network allows for the connection and cross-pollination of multiple professions and areas of expertise for the purpose of discovering existing simulation gaps, providing innovative solutions that change systems, potentially leading to improved healthcare outcomes. Specifically, maxSIMhealth utilizes disruptive technologies, including, but not limited to, 3D printing, gaming, and extended reality, as innovative solutions that allow for cost-effective, portable, and realistic simulation. Thus, it has the potential to provide health professionals with access to simulation . Since simulation is a relatively newer component of education and training practice, it is oftentimes not readily accepted into curriculums due to the notion that it is an additional and resource-demanding task [2]. However, with its improving usability and effectiveness based on increased satisfaction, knowledge, skills, and practice behaviors, it is now imperative to integrate simulation properly throughout education curriculums (e.g. nursing) [3]. Programs can no longer rely on this 'add-on' notion because simulation opts as a replacement for traditional and often rare clinical experiences, and allows learners to develop skills, clinical reasoning, and care competency [4]. With its foundation in technology, sciences, and professional practice, maxSIMhealth thrives on this growing acceptance and enthusiasm of simulation in health professional education. In doing this, it is able to fulfill its vision of advancing the discovery and application of knowledge that revolutionizes health by providing innovative solutions for simulation training and clinical application.

Methods and implementation

**Figure 2:**
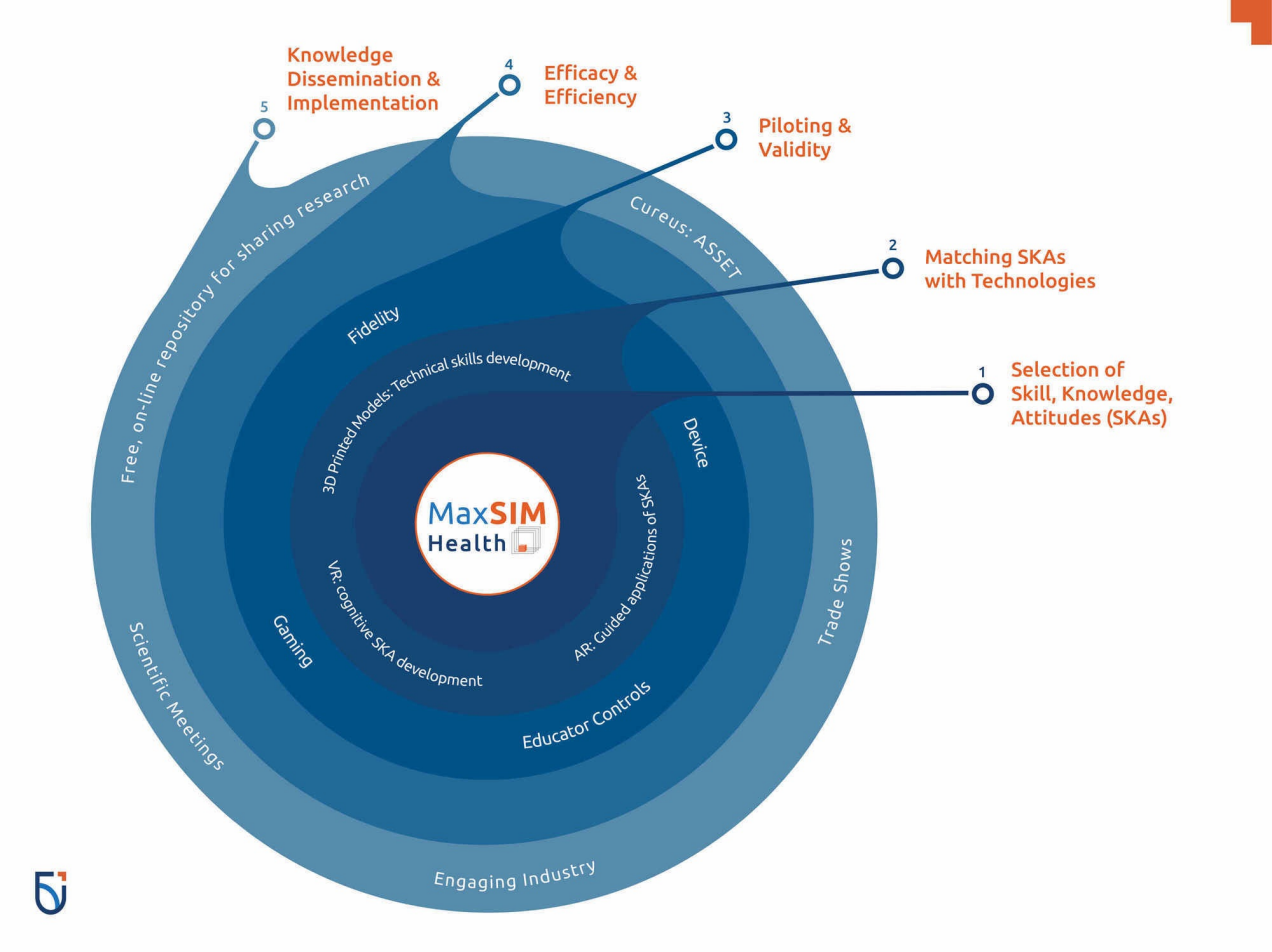
The maxSIMhealth five-step process to develop and translate innovative simulation solutions.

The way maxSIMhealth operates can be summed up by the following three terms: ideate, create, and disseminate. Through this collaborative approach, ideas are taken and transformed into existence, and the final product is disseminated which results in solutions that matter. MaxSIMhealth’s work spans a broad spectrum of scholarship from mapping existing gaps, to changing education systems, to improving learning and performance outcomes. In order to achieve this, the maxSIMhealth map is followed using five simple steps when starting new projects (see Figure 2).

Step 1: Gap Analysis/SKA Selection

This step involves selecting skill, knowledge and/or attitudes (SKA) to focus on and improve through collaboration with content and health professional experts as our partners. A key component of this first step involves the utilization of maxSIMhealth's unique range of established research partnerships with hospitals, professional societies, governing bodies, and the simulation industry. This step follows a methodology adopted from implementation sciences and described in our earlier report [5]. In brief, this step constitutes the formation of core ideas through collaborating with diverse groups of stakeholders outlined above. A needs assessment is a fundamental stage in the educational process, which will lead to changes in practice and therefore, it is the starting point for designing a formalized educational program. The stakeholders’ contextually appropriate ideas are assessed for their ‘fit’ in maxSIMhealth. The needs assessment identifies gaps which must be addressed by looking at the current position of the stakeholders, current curriculum, and comparing it to the desired level of simulation learning. MaxSIMhealth employs diverse methods for conducting a gap analysis: individual interviews, focus groups, surveys, questionnaires, self-assessments, and observations. Next, this information is translated into a detailed implementation plan. The implementation plan addresses the ‘what, who and when’ of the implementation, which identifies activities to be performed, schedules, and people involved. Resources are gathered from internal and external talent to build a functional team, and risks and potential roadblocks are identified [5].

Step 2: Technology Matching

This step is designed to determine the best possible and most feasible disruptive technological solution for the selected SKA. Similar to step 1, this focuses on involving stakeholders and employs diverse methods for conducting a gap analysis: individual interviews, focus groups, surveys, questionnaires, self-assessments, and observations. The step culminates with the formation of development teams including several students, faculty members, and partners. Typical team composition has two students, one technology oriented and one with expertise in health sciences, two faculty advisors one with expertise in health sciences, and at least one partner lead with experience in a health related field (e.g. a medical doctor).

Step 3: Piloting and Validity

Piloting the concept and conducting preliminary studies occurs to determine its face and content validity by testing the realism and educational value, as well as skill learning and retention with experienced practitioners and trainees. 

Step 4: Efficacy and Effectiveness Testing

In this step, we aim to determine whether or not the concept works and, if so, how well it works. In steps 3 and 4, maxSIMhealth follows an adapted Medical Research Council Framework [3]. This work typically constitutes graduate level scholarship, and therefore it adheres to a funding model that emphasizes highly qualified personnel training which must be embedded in all activities. 

Step 5: Knowledge Dissemination and Implementation

This step involves knowledge dissemination of the products and outcomes, as well as implementation into health and/or education systems. To ensure timely and meaningful knowledge translation, maxSIMhealth has also established an institutional channel called Archives of Scholarship in Simulation and Educational Techniques (ASSET) with the open-access Cureus Journal of Medical Science through which our work is freely disseminated as peer-reviewed, PubMed-indexed publications. MaxSIMhealth works with our research partners to distribute the solutions for free, at cost, or at an affordable price point. To accomplish this, we are encouraging institutions to become members with maxSIMhealth and participate in further implementation and iterative improvement research.

With this five-step map (see Figure 2), students and experts within the collaborative work together and follow a set of guidelines that facilitate the development and implementation of simulation solutions that could improve healthcare outcomes.

Conclusions

MaxSIMhealth is a novel and innovative network at Ontario Tech University, straddling many professions and settings. Keeping the goals of public health in mind, we collectively aim to develop future cohorts of scholars who will have strong competencies, ranging from technology application, to working with others in new environments, to communicating professionally and problem-solving. It is anticipated that our work will successfully transform the current health professional education landscape by providing novel, flexible, and potentially cost-effective simulation experiences.
